# Trends in the utilisation of skilled birth attendance among pregnant women in Benin, from 2001 to 2017-2018, and projections to 2030

**DOI:** 10.1186/s12889-023-15460-x

**Published:** 2023-05-12

**Authors:** Pascaline Yvonne Talon, Jacques Saizonou, Alphonse Kpozèhouen, Robert Franck Zannou, Edgard-Marius Ouendo

**Affiliations:** 1 Regional Public Health Institute, University of Abomey-Calavi, BP 384 – Road of slaves, Ouidah, Benin; 2 University Hospital of Mother-Child Lagune of Cotonou, Cotonou, 01 BP 107 Bénin

**Keywords:** Sustainable Development Goals, Women, Skilled birth attendance, Projection, 2030, DHS, Benin

## Abstract

**Background:**

Skilled birth attendance during childbirth is known to promote better pregnancy outcomes as well as contribute to maternal and newborn survival. The study aimed to analyse the progress in the use of skilled birth attendance by pregnant women over the last two decades (2001 to 2017–2018) in Benin, and then to make projections to 2030.

**Methods:**

A secondary analysis was made using Benin's Demographic and Health Survey (DHS) databases. The study population were i) women of 15–49 years of age who were successfully surveyed and usually resided in the households visited during DHS-II, DHS-III, DHS-IV and DHS-V, ii) and had had at least one live birth in the five years preceding each of these surveys. For each DHS, the corresponding proportion of births attended by skilled health personnel was determined. The study then generated the Annual Percent Change (APC) between each survey and globally, and projections were made to 2030.

**Results:**

Nationally, the percentage of women who gave birth attended by skilled health personnel was 67.39% in 2001, 76.10% in 2006, 80.87% in 2011–2012, and 79.12% in 2017–2018; this represents an APC = 0.98% between 2001 and 2017–2018. If the historical rate of progression is maintained, it is expected that by 2030, 89.35% of pregnant women will be using skilled birth attendance services.

**Conclusion:**

Efforts are needed to understand the drivers of skilled birth attendance among pregnant women to adopt appropriate strategies.

## Background

Skilled birth attendance refers to the presence of a trained health professional, such as a midwife, doctor, or nurse, during childbirth. It promotes better pregnancy outcomes and contributes to maternal and newborn survival [1–5]. Over the past two decades, the changing context of skilled birth attendance has been marked by the adoption of the Millennium Development Goals (MDGs) in 2000 and the Sustainable Development Goals (SDGs) in 2015 [6, 7]. The MDGs are a set of eight goals, with 20 targets, monitored by indicators, that United Nations (UN) Member States agreed to strive to achieve by 2015. In particular, target 5.A of MDG5 called on states to improve maternal health, reducing the maternal mortality ratio by three-quarters between 1990 and 2015 [6]. Achievement of this target was monitored through the maternal mortality ratio and the proportion of births attended by skilled health personnel. This reflects the value placed on the use of skilled birth attendance services. Over the MDGs period, the worldwide proportion of births attended by skilled health personnel increased from 59% around 1990 to 71% around 2014 [8]. Meanwhile, globally, the maternal mortality ratio decreased by 45% between 1990 and 2013, from 380 to 210 deaths per 100,000 live births [8]. Moreover, behind the global coverage figures, regional disparities existed in the level of utilisation of skilled birth attendance services. Coverage ranged from universal or near-universal in East and Central Asia to about 52% in sub-Saharan Africa and South Asia, where 86% of maternal deaths worldwide were recorded in 2013 [8]. In Benin, the proportion of births attended by skilled health personnel during the MDGs period increased from 65.5% in 2001 to 77.2% in 2014 [9]. This implies that more than one in five mothers and their infants remained without critical medical care during childbirth.

The SDGs were agreed in September 2015 following the conclusion of the Millennium Agenda [7]. The new agenda identified 17 SDGs and 169 targets for 2030, including for maternal and child health [7]. In particular, target 3.1 of SDG3 called on states to reduce the global maternal mortality ratio to below 70 deaths per 100,000 live births [7]. The report also states that no country should have a maternal mortality rating twice the world mean [7]. Achieving this target should require increasing the coverage of skilled birth attendance services nationwide, while taking into account disparities related to the basic characteristics of pregnant women. Moreover, the proportion of births attended by skilled health personnel continues to be proposed as a key indicator for monitoring target 3.1 of SDG3. Unlike during the MDGs period, a target was not suggested in the new 2030 agenda in terms of the proportion of births attended by skilled health personnel in order to achieve the expected mortality ratio level. No research has been done in the literature to answer this question in Benin. Nevertheless, results from a 2014 study suggested that a decrease in the maternal mortality ratio of one death per 100,000 live births was associated, with an increase in the percentage of births attended by skilled health personnel of 0.066 points [10]. With a maternal mortality ratio of 347 deaths per 100,000 live births towards the end of the MDG period, Benin's achievement of SDG3 target 3.1 should require an increase of nearly 20% in the proportion of births attended by skilled health personnel, i.e., near universal coverage [11]. It should be recalled that in 2018, Benin adopted the operational plan to reduce maternal and neonatal mortality, which aimed to increase by 2022, from 77 to 90%, the coverage of skilled birth attendance nationwide [9].

Based on the results of a national household survey in 2018, three years after the adoption of the SDGs, few changes were observed in the proportion of births attended by skilled health personnel, compared to 78% at the end of the SDGs [12]. With its current rate of progress, is Benin on track to meet SDG3 target 3.1 with a substantial increase in skilled birth attendance in the coming years? To provide some insight into this question, this study analyses the progress in the proportion of births attended by skilled health personnel over the past two decades (2001 to 2017–2018), and makes projections for the coming years. Projections of the prevalence of health problems are useful for informing policymakers about future trends and serving as a basis for advocacy.

## Methods

### Study setting

Benin is a West African country of 114,763 km^2^. Administratively, Benin has 12 departments since the law N° 97–028 of January 15, 1999, on the organization of the territorial administration of the Republic of Benin, namely: Alibori, Atacora, Atlantique, Borgou, Collines, Couffo, Donga, Littoral, Mono, Ouémé, Plateau and Zou [13]. The Beninese population increased from 6,769,914 in 2002 to 10,008,749 in 2013, an annual growth rate of 3.5 per cent [14, 15]. The latest projections from the National Institute of Statistics and Demography (INStaD, formerly the National Institute of Statistics and Economic Analysis) gave a population of 12,535,929 inhabitants in 2021 [16]. In the same year, women of childbearing age represented approximately 23.58% of the Beninese population [16]. The total fertility rate has not changed significantly since 1996 [12]. The average number of children per woman decreased from 6.0 in 2001 to 5.7 in 2017–2018 [12, 17].

### Study type and data source

In this cross-sectional study, secondary analyses were made using databases from the Demographic and Health Surveys (DHS) in Benin. The DHS program provides free access to full survey data files for academic research. Registration is required, after which a request allows the desired databases for downloading, via the website https://dhsprogram.com/. The DHSs are a series of surveys that aim to produce demographic and health indicators from nationally representative samples [18]. In Benin, the DHSs have been conducted by the National Institute of Statistics and Demography (formerly the National Institute of Statistics and Economic Analysis) in collaboration with the Ministry of Health and with the support of Technical and Financial Partners. In total, Benin has conducted five DHSs: DHS-I in 1996, DHS-II in 2001, DHS-III in 2006, DHS-IV in 2011–2012 and DHS-V in 2017–2018.

### Study population

The study population were i) women of 15–49 years of age who were successfully surveyed and usually resided in the households visited during DHS-II (2001), DHS-III (2006), DHS-IV (2011–2012) and DHS-V (2017–2018), ii) and had had at least one live birth in the five years preceding each of these surveys. Women surveyed in DHS-I were not included, as this survey took place before the MDG period, and some variables of interest were not recorded.

### Sampling

The various surveys whose data were used relied on nationally representative samples of women aged 15–49 years obtained through a stratified two-stage random survey. Full details of the sampling designs for DHS-II, DHS-III, DHS-IV, and DHS-V are presented in the full reports of these surveys [12, 17, 19, 20]. The numbers of women aged 15–49 years eligible and successfully interviewed in the different surveys are presented in Table 1 below. A total of 3,415, 10,356, 8,943, and 8,843 of the successfully surveyed women met the selection criteria and were included in the study for DHS-II, DHS-III, DHS-IV, and DHS-V, respectively.

**Table 1:** Number of women aged 15–49 eligible and successfully surveyed in DHS-II, DHS-III, DHS-IV and DHS-V

Items	DHS-II	DHS-III	DHS-IV	DHS-V
	**2001**	**2006**	**2011–2012**	**2017–2018**
Number of households surveyed	5,769	17,511	17,422	14,156
Number of eligible women	6,448	18,851	17,329	16,233
Number of women surveyed	6,219	17,794	16,599	15,928
Response rate (%)	96	96	96	98

### Variables

The dependent variable was skilled birth attendance. It was coded 1 if the last birth attendance was skilled and 0 otherwise. A skilled birth attendance was defined as a birth that was attended by doctors, nurses, or midwives. The remaining variables considered were related to the woman, the household, and the environment. The variables related to the woman include: age, education level, marital status, religion, occupation, exposure to newspapers/magazines, exposure to the radio, exposure to television, health insurance, type of pregnancy, parity, and compliance with antenatal consultations. A woman observed antenatal consultations if she completed at least four, the first of which was in the first trimester of pregnancy. Household variables include: sex of household head, household size, and wealth index. Environmental variables include: area and department. Because of the former administrative division, in the DHS-II, Atacora was defined as Atacora and Donga; Atlantique as Atlantique and Littoral; Borgou as Borgou and Alibori; Mono as Mono and Couffo; Ouémé as Ouémé and Plateau; and Zou as Zou and Collines.

### Data analysis

All calculations took into account the sampling designs of each survey. Stata 15 and Excel were used for data analysis. Participants characteristics were described and compared across surveys using a Chi-2 test. The study generated annual percentage changes (APC) in the proportion of women using skilled birth attendance between each survey and overall. By definition, APC is the percentage increase or decrease in a given parameter between two successive time units (usually the year) over a given period, assuming linearity. In the present study, the time unit considered was the year [21]. The mathematical formula for determining APCs is presented in $\left(\mathrm{a}\right)$. The APCs calculated for the dependent variable were disaggregated by the other study variables. As a result, the proportion of women delivering with skilled birth attendance expected in 2030 was obtained using the equality $\left(\mathrm{b}\right)$ [21, 22].

1 $$\mathrm{APC }\left(\mathrm{\%}\right)= \left[{\left(\frac{{\mathrm{P}}_{{\mathrm{t}}_{2}}}{{\mathrm{P}}_{{\mathrm{t}}_{1}}}\right)}^{\frac{1}{{\mathrm{t}}_{2}-{\mathrm{t}}_{1}}}-1\right]\times 100$$

2 $${\mathrm{P}}_{2030 }={\mathrm{P}}_{2017-18}{\left(1+\mathrm{APC}\right)}^{\mathrm{n}}$$

### Ethical concerns

The study protocol was approved by the internal institutional ethical review board of the Regional Institute of Public Health. The protocols for the selected surveys were approved by the National Health Research Ethics Committee of Benin and the Internal Review Board of ICF (Macro International). Obtaining informed consent from eligible individuals was mandatory. Details of the ethical aspects of the DHS-II, DHS-III, DHS-IV and DHS-V can be found in the full reports of these different surveys [12, 17, 19, 20].

## Results

### Characteristics of study population

Table 2 shows the baseline characteristics of women surveyed in DHS-II, DHS-III, DHS-IV, and DHS-V. We noted that the majority of participants were aged 20–39 years: 84.09% in 2001, 85.68% in 2006, 86.87% in 2011–2012 and 84.98% in 2017–2018. The proportion of women with secondary education and above increased from 8.04% in 2001 to 17.26% in 2017–2018. Regarding marital status, the percentage of women in couples was 95.52% in 2001 compared to 92.60% in 2017–2018. Between 2001 and 2017–2018, about half (47.08% to 55.65%) of the respondents were Christians. The percentage of women engaged in professional activities was as follows: 91.17% in 2001, 87.07% in 2006, 72.20% in 2011–2012 and 83.24% in 2017–2018. Moreover, during the period of interest, there is a decrease in the percentage of women exposed to radio in favor of an increase in the frequency of women exposed to television. Meanwhile, the proportion of women not exposed to newspapers/magazines has remained relatively constant: 93.06% in 2001, 94.66% in 2006, 91.50% in 2011–2012 and 94.24% in 2017–2018. In 2.65–3.09% of the respondents, the last pregnancy was twin. About, 32% of the participants had completed at least four antenatal consultations including the first one in the first trimester in 2001. This proportion increased to 38.10% in 2017–2018. Most of the respondents lived in male households with six or more people. Women living in rural areas were the most represented (59.18% to 66.99%). By departement, women from Zou (20.67%) and Atlantique (19.70%) were the most common in 2001; and those from Alibori (13.61%), Borgou (12.85%) and Atlantique (11.45%) were the most represented in 2017–2018.

**Table 2:** Basic characteristics of women surveyed in DHS-II, DHS-III, DHS-IV and DHS-V, Benin

**Variables**	**DHS-II**		**DHS-III**		**DHS-IV**		**DHS-V**		**p**
	**n**	**%**	**n**	**%**	**n**	**%**	**n**	**%**	
**Total**	3415	100.00	10,356	100.00	8943	100.00	8843	100.00	
**Age**									< 0.001
15–19	181	5.30	497	4.80	371	4.15	474	5.37	
20–29	1695	49.63	5071	48.97	4298	48.06	4363	49.34	
30–39	1177	34.46	3802	36.71	3471	38.81	3151	35.64	
40–49	362	10.61	985	9.52	803	8.98	854	9.65	
**Education level**									< 0.001
Uninstructed	2478	72.57	7589	73.28	6304	70.49	5704	64.50	
Primary	662	19.39	1897	18.32	1496	16.73	1612	18.23	
Secondary and above	275	8.04	870	8.40	1143	12.79	1526	17.26	
**Marital status**									< 0.001
Single	153	4.48	452	4.36	615	6.88	654	7.40	
In couple/dating	3262	95.52	9904	95.64	8328	93.12	8188	92.60	
**Religion**									< 0.001
Traditional and others	699	20.47	2110	20.38	1369	15.31	942	10.65	
Islam	813	23.79	2453	23.68	2134	23.86	2951	33.37	
Christianism	1608	47.08	5175	49.97	4977	55.65	4454	50.37	
No religion	296	8.66	618	5.96	464	5.18	496	5.61	
**Occupation**									< 0.001
No	301	8.83	1339	12.93	2486	27.80	1482	16.76	
Yes	3112	91.17	9015	87.07	6457	72.20	7361	83.24	
**Exposure to newspapers**									< 0.001
Not at all	3168	93.06	9738	94.66	8183	91.50	8334	94.24	
Less than once a week	142	4.18	376	3.65	346	3.87	302	3.42	
At least once a week	94	2.76	173	1.69	414	4.63	207	2.34	
**Exposure to radio**									< 0.001
Not at all	621	18.20	1967	19.03	3431	38.37	3914	44.27	
Less than once a week	638	18.69	2637	25.51	1778	19.88	1874	21.19	
At least once a week	2154	63.11	5731	55.46	3734	41.76	3055	34.55	
**Exposure to TV**									< 0.001
Not at all	2618	76.69	7195	69.88	5076	56.76	5787	65.45	
Less than once a week	279	8.19	1115	10.83	1216	13.59	1447	16.36	
At least once a week	517	15.13	1986	19.29	2651	29.64	1609	18.19	
**Health insurance**									0.008
No					8816	98.58	8766	99.13	
Yes					127	1.42	77	0.87	
**Parity**									< 0.001
1	681	19.95	1791	17.30	1676	18.75	1686	19.07	
2	577	16.90	1853	17.90	1716	19.19	1599	18.08	
3	472	13.81	1669	16.11	1660	18.56	1464	16.55	
4 and more	1685	49.34	5043	48.69	3890	43.50	4094	46.30	
**Type of pregnancy**									0.405
Simple	3317	97.12	10,036	96.91	8706	97.35	8591	97.15	
Twins	98	2.88	320	3.09	237	2.65	252	2.85	
**Antenatal consultations**									0.002
No	2317	67.83	6547	63.22	5528	61.81	5474	61.90	
Yes	1099	32.17	3808	36.78	3415	38.19	3369	38.10	
**Sex of household head**									< 0.001
Male	2989	87.53	8958	86.50	7743	86.58	7385	83.52	
Female	426	12.47	1398	13.50	1201	13.42	1458	16.48	
**Variables**	**DHS-II**	**DHS-III**	**DHS-IV**	**DHS-V**	**p**				
	**n**	**%**	**n**	**%**	**n**	**%**	**n**	**%**	
**Household size**									< 0.001
1–5	1361	39.84	4706	45.44	4011	44.85	3536	39.98	
6 +	2054	60.16	5650	54.56	4932	55.15	5307	60.02	
**Wealth level**									0.778
Poorest			2183	21.08	1803	20.17	1784	20.18	
Poorer			2059	19.88	1794	20.06	1784	20.17	
Middle			2140	20.66	1779	19.90	1801	20.36	
Richer			2135	20.62	1790	20.02	1819	20.57	
Richest			1839	17.76	1776	19.86	1655	18.72	
**Area**									0.045
Urban	1127	33.01	3675	35.49	3651	40.82	3460	39.12	
Rural	2288	66.99	6680	64.51	5292	59.18	5383	60.88	
**Department**									< 0.001
Alibori			930	8.98	597	6.67	1204	13.61	
Atacora	471	13.80	738	7.13	836	9.35	769	8.69	
Atlantique	673	19.70	1151	11.12	1089	12.18	1013	11.45	
Borgou	605	17.71	1011	9.76	685	7.66	1137	12.85	
Collines			744	7.18	582	6.51	592	6.69	
Couffo			851	8.22	649	7.26	579	6.54	
Donga			426	4.11	384	4.30	592	6.69	
Littoral			759	7.33	1059	11.85	399	4.51	
Mono	402	11.76	628	6.06	486	5.43	390	4.41	
Ouémé	558	16.35	1410	13.61	1136	12.70	795	8.99	
Plateau			539	5.20	621	6.94	537	6.07	
Zou	706	20.67	1169	11.29	818	9.15	838	9.48	

### Association between pregnant women's recourse to skilled birth attendance and baseline characteristics

Table 3 shows the association between the basic characteristics of the surveyed women and the use of skilled birth attendance. There is an association between the age of the respondents and the use of skilled birth attendance (*p* < 0.05). In addition, the use of skilled birth attendance was significantly higher among women with secondary education and above, single or Christian (*p* < 0.05). The same was true for women exposed to newspapers, radio and television (*p* < 0.05). Also, the use of skilled birth attendance varied significantly by whether or not they were employed (*p* < 0.05). In addition, respondents with health insurance had more frequent births attended by skilled health personnel compared to those without (*p* < 0.05). Skilled birth attendance was also higher among primiparous women and those attending antenatal consultations (*p* < 0.05). In addition, the proportion of births attended by skilled health personnel was higher in female-headed household, less than six-person, wealthy households (*p* < 0.05). According to the area, the percentage of women who gave birth with the attendance of skilled health personnel was significantly higher in urban areas (*p* < 0.05). Finally, the proportion of births attended by skilled health personnel varied significantly according to the department of the participants (*p* < 0.05).

**Table 3:** Relationship between basic characteristics of the women surveyed and recourse to skilled birth attendance, Benin

**Variables**	**DHS-II**			**DHS-III**			**DHS-IV**			**DHS-V**		
	**n**	**%**	**p**	**n**	**%**	**p**	**n**	**%**	**p**	**n**	**%**	**p**
**Total**	2301	67.39		7881	76.10		7328	80.87		6975	79.12	
**Age**			0.043			0.008			0.298			< 0.001
15–19	119	65.65		358	72.02		307	82.89		374	78.81	
20–29	1150	67.84		3916	77.23		3549	82.57		3464	79.39	
30–39	811	68.95		2891	76.03		2809	80.93		2557	81.15	
40–49	221	61.04		716	72.65		662	82.41		625	73.23	
**Education level**			< 0.001			< 0.001			< 0.001			< 0.001
Uninstructed	1512	61.00		5336	70.31		4823	76.51		4193	73.52	
Primary	529	79.94		1707	89.98		1402	93.75		1424	88.33	
Secondary and above	260	94.75		838	96.36		1102	96.38		1403	91.91	
**Marital status**			< 0.001			0.041			0.293			< 0.001
Single	128	83.85		363	80.46		517	83.96		572	87.43	
In couple/dating	2173	66.61		7517	75.90		6811	81.78		6448	78.75	
**Religion**			< 0.001			< 0.001			< 0.001			< 0.001
Traditional and others	387	55.41		1490	70.60		1035	75.55		732	77.70	
Islam	480	59.13		1485	60.55		1431	67.07		1924	65.20	
Christianism	1257	78.18		4480	86.57		4506	90.54		4039	90.68	
No religion	177	59.71		426	68.91		356	76.76		326	65.68	
**Occupation**			0.030			0.759			< 0.001			< 0.001
No	223	74.16		1012	75.55		1813	72.91		1064	71.80	
Yes	2077	66.75		6867	76.18		5515	85.41		5957	80.92	
**Exposure to newspapers**			< 0.001			< 0.001			< 0.001			< 0.001
Not at all	2069	65.31		7293	74.90		6590	80.53		6562	78.74	
Less than once a week	136	95.89		366	97.23		334	96.42		269	88.97	
At least once a week	87	92.24		167	96.19		404	97.45		190	91.86	
**Exposure to radio**			< 0.001			< 0.001			< 0.001			< 0.001
Not at all	317	51.05		1227	62.38		2545	74.17		2879	73.55	
Less than once a week	393	61.64		1930	73.21		1473	82.86		1574	84.01	
At least once a week	1589	73.77		4709	82.16		3309	88.62		2568	84.05	
**Exposure to TV**			< 0.001			< 0.001			< 0.001			< 0.001
Not at all	1600	61.13		5033	69.96		3707	73.03		4366	75.44	
Less than once a week	223	79.87		943	84.52		1083	89.04		1210	83.64	
At least once a week	477	92.32		1865	93.92		2538	95.73		1445	89.81	
**Health insurance**									0.007			0.016
No							7211	81.79		6950	79.29	
Yes							117	91.96		70	91.76	
**Parity**			< 0.001			< 0.001			< 0.001			< 0.001
1	525	77.07		1496	83.51		1442	86.02		1423	84.42	
2	400	69.26		1491	80.46		1462	85.17		1296	81.04	
3	322	68.19		1320	79.11		1369	82.50		1176	80.34	
4 and more	1055	62.61		3574	70.87		3054	78.50		3126	76.34	
**Variables**	**DHS-II**			**DHS-III**			**DHS-IV**			**DHS-V**		
	**n**	**%**	**p**	**n**	**%**	**p**	**n**	**%**	**p**	**n**	**%**	**p**
**Type of pregnancy**			0.104			< 0.001			< 0.001			0.282
Simple	2228	67.16		7607	75.80		7105	81.62		6814	79.32	
Twins	74	75.09		273	85.49		222	93.48		207	82.03	
**Antenatal consultations**			< 0.001			< 0.001			< 0.001			< 0.001
No	1387	59.88		4400	67.21		4115	74.44		4017	73.39	
Yes	914	83.22		3481	91.39		3212	94.05		3003	89.15	
**Sex of household head**			< 0.001			< 0.001			< 0.001			< 0.001
Male	1953	65.34		6707	74.87		6254	80.78		5782	78.29	
Female	348	81.73		1174	84.01		1073	89.39		1239	84.99	
**Household size**			0.006			< 0.001			< 0.001			< 0.001
1–5	960	70.53		3900	82.87		3498	87.22		3076	87.01	
6 +	1342	65.30		3981	70.47		3829	77.64		3944	74.32	
**Wealth level**						< 0.001			< 0.001			< 0.001
Poorest				1178	53.96		1101	61.06		1033	57.93	
Poorer				1409	68.44		1335	74.42		1330	74.53	
Middle				1640	76.65		1464	82.26		1450	80.54	
Richer				1886	88.35		1692	94.49		1634	89.85	
Richest				1767	96.11		1735	97.73		1573	95.03	
**Area**			< 0.001			< 0.001			< 0.001			< 0.001
Urban	919	81.53		3151	85.73		3322	91.00		2981	86.15	
Rural	1382	60.41		4730	70.80		4005	75.68		4040	75.05	
**Department**						< 0.001			< 0.001			< 0.001
Alibori		54.67	< 0.001	432	46.38		248	41.56		605	50.27	
Atacora	222	47.08		364	49.30		544	65.03		517	67.20	
Atlantique	600	89.19		1114	96.73		1001	91.94		957	94.48	
Borgou	331	54.67		571	56.50		477	69.60		735	64.66	
Collines		68.44		556	74.78		517	88.81		554	93.61	
Couffo		54.26		596	70.02		525	80.87		457	79.02	
Donga		47.08		288	67.50		273	71.10		474	80.16	
Littoral		89.19		735	96.83		1035	97.67		371	92.97	
Mono	218	54.26		551	87.73		434	89.34		333	85.31	
Ouémé	448	80.16		1289	91.42		1105	97.25		775	97.48	
Plateau		80.16		406	75.31		396	63.74		494	92.02	
Zou	483	68.44		981	83.91		773	94.48		749	89.38	

### Trend in the use of skilled birth attendance

Nationally, the percentage of women who gave birth with the attendance of skilled health personnel increased from 67.39% in 2001 to 79.12% in 2017–2018, or an APC = 0.98% (Table 4). The largest relative increase was observed between 2001 and 2006, with an APC = 2.46%. During the period of interest and according to the basic characteristics of the respondents, APCs higher than two percentage points were recorded in Donga (APC = 3.28%), Mono (APC = 2.78%), Atacora (APC = 2.18%), as well as among women of traditional religion and others (APC = 2.07), not exposed to radio (APC = 2.24%).

**Table 4:** APC of the use of skilled birth attendance among women surveyed in DHS-II, DHS-III, DHS-IV and DHS-V, Benin

Variables	2001 to 2006	2006 to 2011–12	2011–12 to 2017–18	2001 to 2017–18
**Total**	2.46	1.11	-0.36	0.98
**Age**				
15–19	1.87	2.59	-0.84	1.11
20–29	2.63	1.22	-0.65	0.96
30–39	1.97	1.14	0.05	0.99
40–49	3.54	2.32	-1.95	1.11
**Education level**				
Uninstructed	2.88	1.55	-0.66	1.14
Primary	2.39	0.75	-0.99	0.61
Secondary and above	0.34	0.01	-0.79	-0.18
**Marital status**				
Single	-0.82	0.78	0.68	0.25
In couple/dating	2.64	1.37	-0.63	1.02
**Religion**				
Traditional and others	4.96	1.24	0.47	2.07
Islam	0.48	1.88	-0.47	0.59
Christianism	2.06	0.82	0.03	0.90
No religion	2.91	1.98	-2.57	0.58
**Occupation**				
No	0.37	-0.64	-0.26	-0.20
Yes	2.68	2.10	-0.89	1.17
**Exposure to newspapers**				
Not at all	2.78	1.33	-0.38	1.14
Less than once a week	0.28	-0.15	-1.33	-0.45
At least once a week	0.84	0.24	-0.98	-0.03
**Exposure to radio**				
Not at all	4.09	3.20	-0.14	2.24
Less than once a week	3.50	2.28	0.23	1.89
At least once a week	2.18	1.39	-0.88	0.79
**Exposure to TV**				
Not at all	2.74	0.78	0.54	1.28
Less than once a week	1.14	0.95	-1.04	0.28
At least once a week	0.35	0.35	-1.06	-0.17
**Health insurance**				
No			-0.52	-0.52
Yes			-0.04	-0.04
**Parity**				
1	1.62	0.54	-0.31	0.55
2	3.04	1.04	-0.82	0.96
3	3.01	0.77	-0.44	1.00
4 and more	2.51	1.88	-0.46	1.21
**Variables**	**2001 to 2006**	**2006 to 2011–12**	**2011–12 to 2017–18**	**2001 to 2017–18**
**Type of pregnancy**				
Simple	2.45	1.35	-0.48	1.01
Twins	2.63	1.64	-2.15	0.54
**Antenatal consultations**				
No	2.34	1.88	-0.24	1.24
Yes	1.89	0.52	-0.89	0.42
**Sex of household head**				
Male	2.76	1.39	-0.52	1.10
Female	0.55	1.13	-0.84	0.24
**Household size**				
1–5	3.28	0.93	-0.04	1.28
6 +	1.53	1.78	-0.72	0.79
**Wealth level**				
Poorest		2.27	-0.88	0.43
Poorer		1.53	0.03	0.52
Middle		1.29	-0.35	0.30
Richer		1.23	-0.84	0.10
Richest		0.30	-0.47	-0.07
**Area**				
Urban	1.01	1.09	-0.91	0.33
Rural	3.22	1.22	-0.14	1.32
**Department**				
Alibori	-3.23	-1.98	3.22	-0.51
Atacora	0.92	5.16	0.55	2.18
Atlantique	1.64	-0.92	0.46	0.35
Borgou	0.66	3.86	-1.22	1.02
Collines	1.79	3.18	0.88	1.92
Couffo	5.23	2.65	-0.39	2.30
Donga	7.47	0.95	2.02	3.28
Littoral	1.66	0.16	-0.82	0.25
Mono	10.09	0.33	-0.77	2.78
Ouémé	2.66	1.13	0.04	1.19
Plateau	-1.24	-2.99	6.31	0.84
Zou	4.16	2.18	-0.92	1.63

### Projections to 2030

**Fig. 1:**
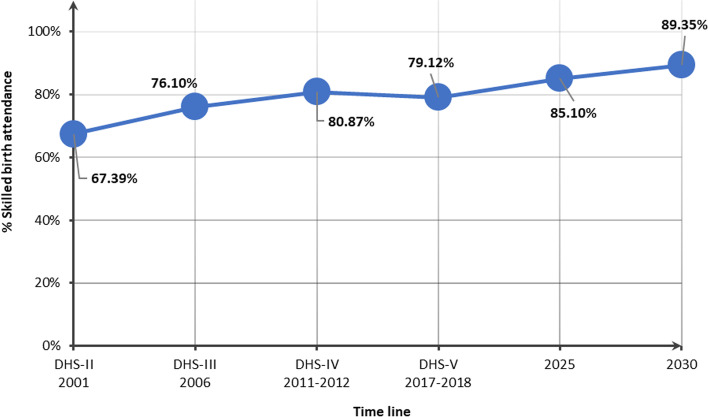
Proportion of births attended by skilled health personnel in Benin from 2001 to 2017–2018, and projections to 2030

If the historical rate of increase is maintained, the proportion of births attended by skilled health personnel.is expected to reach 89.35% by 2030 (Fig. 1).

Table 5 describes the projected utilisation of skilled birth attendance by pregnant women from 2019 to 2030. A universal coverage in skilled birth attendance is projected for women with secondary education and above, of Christian faith, exposed to newspapers/magazines or television and observing antenatal consultations. Also, skilled birth attendance coverage is planned for the Atlantique, Collines, Littoral, Ouémé, Plateau and Zou regions.

**Table 5:** Projections (%) of pregnant women's use of skilled birth attendance from 2019 to 2030, Benin

Variables	Projections											
	**2019**	**2020**	**2021**	**2022**	**2023**	**2024**	**2025**	**2026**	**2027**	**2028**	**2029**	**2030**
**Total**	80.28	81.06	81.86	82.66	83.47	84.28	85.10	85.94	86.78	87.62	88.48	89.35
**Age**												
15–19	79.97	80.75	81.54	82.34	83.14	83.96	84.78	85.61	86.44	87.29	88.14	89.00
20–29	80.56	81.35	82.14	82.95	83.76	84.58	85.40	86.24	87.08	87.93	88.79	89.66
30–39	82.35	83.15	83.96	84.78	85.61	86.45	87.29	88.15	89.01	89.88	90.76	91.64
40–49	74.30	75.03	75.76	76.50	77.25	78.01	78.77	79.54	80.32	81.10	81.89	82.69
**Education level**												
Uninstructed	74.60	75.33	76.06	76.81	77.56	78.32	79.08	79.85	80.63	81.42	82.22	83.02
Primary	89.63	90.51	91.39	92.28	93.19	94.10	95.02	95.95	96.88	97.83	98.79	> 99.00
Secondary and above	93.26	94.17	95.09	96.02	96.96	97.91	98.87	> 99.00	> 99.00	> 99.00	> 99.00	> 99.00
**Marital status**												
Single	88.72	89.59	90.46	91.35	92.24	93.14	94.05	94.97	95.90	96.84	97.78	98.74
In couple/dating	79.91	80.69	81.48	82.27	83.08	83.89	84.71	85.54	86.38	87.22	88.07	88.93
**Religion**												
Traditional and others	78.84	79.61	80.39	81.17	81.96	82.77	83.57	84.39	85.22	86.05	86.89	87.74
Islam	66.16	66.80	67.46	68.12	68.78	69.45	70.13	70.82	71.51	72.21	72.92	73.63
Christianism	92.02	92.92	93.82	94.74	95.67	96.60	97.55	98.50	> 99.00	> 99.00	> 99.00	> 99.00
No religion	66.64	67.29	67.95	68.62	69.29	69.96	70.65	71.34	72.04	72.74	73.45	74.17
**Occupation**												
No	72.85	73.56	74.28	75.01	75.74	76.48	77.23	77.98	78.75	79.52	80.29	81.08
Yes	82.11	82.92	83.73	84.54	85.37	86.21	87.05	87.90	88.76	89.63	90.50	91.39
**Exposure to newspapers**												
Not at all	79.89	80.68	81.46	82.26	83.06	83.88	84.70	85.52	86.36	87.20	88.06	88.92
Less than once a week	90.28	91.16	92.05	92.95	93.86	94.78	95.71	96.64	97.59	98.54	> 99.00	> 99.00
At least once a week	93.21	94.12	95.04	95.97	96.90	97.85	98.81	> 99.00	> 99.00	> 99.00	> 99.00	> 99.00
**Exposure to radio**												
Not at all	74.63	75.36	76.09	76.84	77.59	78.35	79.11	79.89	80.67	81.46	82.25	83.06
Less than once a week	85.25	86.08	86.92	87.77	88.63	89.49	90.37	91.25	92.14	93.05	93.95	94.87
At least once a week	85.29	86.12	86.96	87.81	88.67	89.54	90.41	91.30	92.19	93.09	94.00	94.92
**Exposure to TV**												
Not at all	76.55	77.29	78.05	78.81	79.58	80.36	81.15	81.94	82.74	83.55	84.37	85.19
Less than once a week	84.87	85.70	86.53	87.38	88.23	89.10	89.97	90.85	91.74	92.63	93.54	94.45
At least once a week	91.13	92.02	92.92	93.83	94.74	95.67	96.61	97.55	98.50	> 99.00	> 99.00	> 99.00
**Health insurance**												
No	80.45	81.24	82.03	82.83	83.64	84.46	85.29	86.12	86.96	87.81	88.67	89.54
Yes	93.11	94.02	94.94	95.87	96.80	97.75	98.70	> 99.00	> 99.00	> 99.00	> 99.00	> 99.00
**Parity**												
1	85.66	86.50	87.35	88.20	89.06	89.93	90.81	91.70	92.60	93.50	94.41	95.34
2	82.23	83.04	83.85	84.67	85.50	86.33	87.18	88.03	88.89	89.76	90.64	91.52
3	81.52	82.32	83.12	83.93	84.75	85.58	86.42	87.26	88.12	88.98	89.85	90.73
4 +	77.46	78.22	78.98	79.76	80.54	81.32	82.12	82.92	83.73	84.55	85.38	86.21
**Variables**	**Projections**											
	**2019**	**2020**	**2021**	**2022**	**2023**	**2024**	**2025**	**2026**	**2027**	**2028**	**2029**	**2030**
**Type of pregnancy**												
Simple	80.48	81.27	82.06	82.87	83.68	84.49	85.32	86.15	87.00	87.85	88.70	89.57
Twins	83.24	84.05	84.87	85.70	86.54	87.39	88.24	89.10	89.97	90.85	91.74	92.64
**Antenatal consultations**												
No	74.47	75.19	75.93	76.67	77.42	78.18	78.94	79.71	80.49	81.28	82.07	82.88
Yes	90.46	91.35	92.24	93.14	94.05	94.97	95.90	96.84	97.78	98.74	> 99.00	> 99.00
**Sex of household head**												
Male	79.44	80.22	81.00	81.79	82.59	83.40	84.21	85.04	85.87	86.71	87.55	88.41
Female	86.24	87.09	87.94	88.80	89.66	90.54	91.43	92.32	93.22	94.13	95.05	95.98
**Household size**												
1–5	88.29	89.15	90.02	90.90	91.79	92.69	93.59	94.51	95.43	96.37	97.31	98.26
6 +	75.41	76.15	76.89	77.65	78.40	79.17	79.94	80.73	81.52	82.31	83.12	83.93
**Wealth level**												
Poorest	58.78	59.35	59.93	60.52	61.11	61.71	62.31	62.92	63.53	64.15	64.78	65.41
Poorer	75.63	76.37	77.11	77.87	78.63	79.40	80.17	80.96	81.75	82.55	83.35	84.17
Middle	81.73	82.53	83.33	84.15	84.97	85.80	86.64	87.49	88.34	89.20	90.08	90.96
Richer	91.17	92.06	92.96	93.87	94.79	95.71	96.65	97.59	98.55	> 99.00	> 99.00	> 99.00
Richest	96.43	97.37	98.32	> 99.00	> 99.00	> 99.00	> 99.00	> 99.00	> 99.00	> 99.00	> 99.00	> 99.00
**Area**												
Urban	87.42	88.28	89.14	90.01	90.89	91.78	92.67	93.58	94.49	95.42	96.35	97.29
Rural	76.15	76.90	77.65	78.41	79.17	79.95	80.73	81.52	82.31	83.12	83.93	84.75
**Department**												
Alibori	51.01	51.51	52.02	52.52	53.04	53.56	54.08	54.61	55.14	55.68	56.22	56.77
Atacora	68.18	68.85	69.52	70.20	70.89	71.58	72.28	72.99	73.70	74.42	75.15	75.88
Atlantique	95.87	96.81	97.76	98.71	> 99.00	> 99.00	> 99.00	> 99.00	> 99.00	> 99.00	> 99.00	> 99.00
Borgou	65.61	66.25	66.90	67.55	68.21	68.88	69.55	70.23	70.92	71.61	72.31	73.02
Collines	94.98	95.91	96.85	97.80	98.75	> 99.00	> 99.00	> 99.00	> 99.00	> 99.00	> 99.00	> 99.00
Couffo	80.18	80.96	81.75	82.55	83.36	84.17	85.00	85.83	86.67	87.51	88.37	89.23
Donga	81.34	82.13	82.94	83.75	84.56	85.39	86.23	87.07	87.92	88.78	89.65	90.52
Littoral	94.33	95.25	96.18	97.13	98.07	> 99.00	> 99.00	> 99.00	> 99.00	> 99.00	> 99.00	> 99.00
Mono	86.56	87.41	88.26	89.13	90.00	90.88	91.77	92.66	93.57	94.48	95.41	96.34
Ouémé	98.91	> 99.00	> 99.00	> 99.00	> 99.00	> 99.00	> 99.00	> 99.00	> 99.00	> 99.00	> 99.00	> 99.00
Plateau	93.38	94.29	95.21	96.14	97.08	98.03	98.99	> 99.00	> 99.00	> 99.00	> 99.00	> 99.00
Zou	90.69	91.58	92.48	93.38	94.29	95.21	96.15	97.08	98.03	98.99	> 99.00	> 99.00

## Discussion

The present study aimed to analyse the progression of the utilisation of skilled birth attendance over the last two decades in Benin and to make projections for the coming years.

It was noted that nationally, the percentage of women who delivered with skilled birth attendance increased from 67.39% in 2001 to 79.12% in 2017–2018. This represents an absolute gain of about 12 percentage points. At the strategic level, this progress can be attributed to a government commitment to maternal, newborn and child health. In the health sector, this commitment has, among other things, resulted in the adoption and implementation of policies, health development plans, and strategies that make maternal health a priority for the health system [23–25]. There has also been an increase in the availability of skilled health professionals in health facilities and an improvement in the geographic accessibility of health facilities. Indeed, at the national level, the average theoretical radius of action of health facilities has decreased from 7.8 in 2007 to 5.2 in 2018 [26, 27]. We should note that, although efforts have been made in terms of the availability of human resources in health, current levels remain below the standards set by the WHO [16, 26, 28]. In addition, the gain we observed could also be related to favourable changes in socio-demographic and behavioural factors that influence the use of skilled birth attendance by pregnant women. In particular, some studies have found a positive and significant relationship between the utilisation of skilled birth attendance by pregnant women and their level of education and compliance with antenatal consultations, respectively [29–33]. We found that the proportion of women with secondary education and above increased twofold, from 8.04% to 17.26%. At the same time, compliance with antenatal consultations has been strengthened (32.17% in 2001 to 38.10% in 2017–2018). These factors, which are not exhaustive, has contributed in varying degrees to the improvement in coverage in the use of skilled professionals during childbirth. Over the reporting period, progress was also observed in other countries, regions, and globally. Globally, the percentage of births attended by skilled health personnel increased from 64% in 2001–2007 to 84% in 2015–2021 [34]. In Sub-Saharan Africa, over the same period, the indicator rose from 43 to 64% [34]. In other regions, namely Europe, North America and Central Asia, near-universal or universal coverage was recorded over the period of interest [34]. The study also highlighted that the growth observed has not been constant; the largest relative gain was found between 2001 and 2006.

The results of this study also include a disaggregated analysis by basic characteristics, highlighting some inequalities. The study found significant variation in the utilisation of skilled birth attendance by basic characteristics of pregnant women. It should be recalled that in the present study, the search for associations between baseline characteristics of pregnant women and the utilisation of skilled birth attendance was limited to a univariate analysis. Overall, the associations found were consistent with the findings of other studies [29–33, 35–37]. Subject to multivariate analysis, the found associations reflect the multifactorial nature of the issue of skilled birth attendance, indicating that remedial actions should be targeted to specific groups. In particular, we found that the utilisation of skilled birth attendance was significantly lower among women with no formal education, no employment, no exposure to media (newspapers/magazines, radio, and television), no health insurance, no antenatal consultation attendance, poor and rural. Further studies are needed to increase knowledge and understanding of the drivers of skilled birth attendance services using multivariate techniques.

By the year 2030, Benin is not projected to be on track to achieve universal utilisation of skilled birth attendance by pregnant women. Indeed, if the historical progress is maintained, the expected utilisation of skilled birth attendance services is expected to be 89.35%, with some disparities related to the basic characteristics of pregnant women. In the literature, few studies have attempted to project the percentage of births attended by skilled personnel. One study in 2022 suggested that coverage projections among 15–19 years old women for some African countries, including Angola, Central African Republic, Chad, Ethiopia, Guinea, Madagascar, Niger, Nigeria, Senegal, and Togo, will remain below 80% in 2030 [38]. According to these authors, the policies and programs implemented in countries that experienced the greatest advancements in health coverage can serve as models for improving health care coverage in countries that still lag behind [38].

Our study had strengths and limitations. This study is the first of its kind to examine this issue. Another strength of this work is the use of data from several household surveys, which were nationally representative of the Beninese population and based on similar protocols, making the comparisons that were made relevant. As mentioned above, the findings can be used to inform policymakers about future trends and serve as a basis for advocacy. There are a few limitations to this study. As the dependent variables were filled in on the basis of the respondents' declarations, an information bias cannot be ruled out, especially as a post verification was not carried out. It should be noted that the constraints linked to the variables available in the databases used did not allow us to highlight other factors of heterogeneity in the results found at the national level. Furthermore, the calculation of the APCs is based on the assumption of linearity, which means that it is possible that the projections made may be overestimated or underestimated if the national context is significantly different from what has been observed over the last twenty years.

## Conclusion

Benin has made substantial progress in offering skilled assistance to pregnant women during childbirth. However, behind the national coverage level there are disparities related to the basic characteristics of pregnant women. Thus, if the historical progress is maintained, the country is not on track to ensure universal access to skilled birth attendance for pregnant women by 2030. Therefore, efforts are needed to understand the drivers of skilled birth attendance among pregnant women to adopt appropriate strategies.

## Data Availability

The database we used can be downloaded after a request via www.dhsprogram.com.

## Abbreviations

APC: Annual Percent Change
DHS: Demographic and Health Survey
INStaD: National Institute of Statistics and Demography
MDG: Millennium Development Goals
SDG: Sustainable Development Goals
UN: United Nations
WHO: World Health Organization

## References

[1] Yakoob MY, Ali MA, Ali MU, Imdad A, Lawn JE, Van Den Broek N (2011). The effect of providing skilled birth attendance and emergency obstetric care in preventing stillbirths. BMC Public Health.

[2] Independent Evaluation Group (2016). Delivering the Millennium Development Goals to Reduce Maternal and Child Mortality: A Systematic Review of Impact Evaluation Evidence.

[3] World Health Organization, International Confederation of Midwives, Fédération internationale de Gynécologie et d’Obstétrique (2004). Making pregnancy safer : the critical role of the skilled attendant : a joint statement by WHO, ICM and FIGO.

[4] Montgomery AL, Fadel S, Kumar R, Bondy S, Moineddin R, Jha P (2014). The Effect of Health-Facility Admission and Skilled Birth Attendant Coverage on Maternal Survival in India: A Case-Control Analysis. PLoS ONE.

[5] Singh K, Brodish P, Suchindran C (2014). A Regional Multilevel Analysis: Can Skilled Birth Attendants Uniformly Decrease Neonatal Mortality?. Matern Child Health J.

[6] United Nations Development Group (2003). Indicators for Monitoring the Millennium Development Goals.

[7] United Nations General Assembly (2017). Resolution adopted by the General Assembly on 6 July 2017.

[8] Organisation des Nations Unies (2015). Rapport 2015: Objectifs du Millénaire pour le développement.

[9] de Santé M (2018). Plan opérationnel de réduction de la mortalité maternelle et néonatale au Bénin.

[10] Berhan Y, Berhan A (2014). Skilled Health Personnel Attended Delivery as a Proxy Indicator for Maternal and Perinatal Mortality: A Systematic Review. Ethiop J Health Sci.

[11] Institut National de la Statistique et de l’Analyse Économique (2014). Rapport final - Enquête par grappes à indicateurs multiples (MICS).

[12] Institut National de la Statistique et de l’Analyse Economique, ICF (2019). Enquête Démographique et de Santé 2017–2018.

[13] République du Bénin (1999). Loi n°97–028 du 15 janvier 1999 portant organisation de l’administration territorial.

[14] Institut National de la statistique et de l’analyse économique (2015). RGPH-4 : que retenir des effectifs de population en 2013.

[15] Institut National de la Statistique et de l’Analyse Économique (2016). Recensement Général de la Population et de l’Habitation IV : Cahier des villages et quartiers de ville Littoral.

[16] de Santé M (2022). Annuaire des statistiques sanitaires 2021.

[17] Institut National de la Statistique et de l’Analyse Economique, ORC Macro (2001). Enquête Démographique et de Santé 2001.

[18] Croft, Trevor N, Aileen MJM, Courtney KA (2018). Guide to DHS Statistics.

[19] Institut National de la Statistique et de l’Analyse Economique, Macro International Inc (2007). Enquête Démographique et de Santé 2006.

[20] Institut National de la Statistique et de l’Analyse Economique (2013). Enquête Démographique et de Santé IV.

[21] Fay MP, Tiwari RC, Feuer EJ, Zou Z (2006). Estimating average annual percent change for disease rates without assuming constant change. Biometrics.

[22] Johri M, Subramanian SV, Koné GK, Dudeja S, Chandra D, Minoyan N (2016). Maternal Health Literacy Is Associated with Early Childhood Nutritional Status in India. J Nutr.

[23] de la Santé (Bénin) M (2009). Politique Nationale de Santé.

[24] de la Santé (Bénin) M (2009). Plan National de Développement Sanitaire 2009–2017.

[25] Gbangbade S (2006). Stratégie nationale de réduction de la mortalité maternelle et néonatale 2006–2015.

[26] de la Santé M (2019). Annuaire des Statistiques Sanitaires 2018.

[27] de la Santé M (2008). Annuaire des Statistiques Sanitaires 2007.

[28] de la Santé (Bénin) M (2018). Annuaire des Statistiques Sanitaires 2017.

[29] Seidu A-A, Ahinkorah BO, Agbaglo E, Oduro JK, Amoah A, Yaya S (2022). Factors associated with the utilisation of skilled delivery services in Papua New Guinea: evidence from the 2016–2018 Demographic and Health Survey. Int Health.

[30] Yaya S, Zegeye B, Ahinkorah BO, Seidu A-A, Ameyaw EK, Adjei NK (2021). Predictors of skilled birth attendance among married women in Cameroon: further analysis of 2018 Cameroon Demographic and Health Survey. Reprod Health.

[31] Ameyaw EK, Dickson KS (2020). Skilled birth attendance in Sierra Leone, Niger, and Mali: analysis of demographic and health surveys. BMC Public Health.

[32] Tessema ZT, Tesema GA (2020). Pooled prevalence and determinants of skilled birth attendant delivery in East Africa countries: a multilevel analysis of Demographic and Health Surveys. Ital J Pediatr.

[33] Kibria GMA, Ghosh S, Hossen S, Barsha RAA, Sharmeen A, Uddin SMI (2017). Factors affecting deliveries attended by skilled birth attendants in Bangladesh. Matern Health Neonatol Perinatol.

[34] United Nations Children’s Fund, World Health Organization S (2022). Births attended by skilled health personnel (%) - Joint UNICEF/WHO database.

[35] Mezmur M, Navaneetham K, Letamo G, Bariagaber H (2017). Individual, household and contextual factors associated with skilled delivery care in Ethiopia: Evidence from Ethiopian demographic and health surveys. PLoS ONE.

[36] Samuel O, Zewotir T, North D (2021). Decomposing the urban–rural inequalities in the utilisation of maternal health care services: evidence from 27 selected countries in Sub-Saharan Africa. Reprod Health.

[37] Saaka M, Akuamoah-Boateng J (2020). Prevalence and Determinants of Rural-Urban Utilization of Skilled Delivery Services in Northern Ghana. Scientifica.

[38] Rahman MdM, Taniguchi H, Nsashiyi RS, Islam R, Mahmud SR, Rahman S (2022). Trend and projection of skilled birth attendants and institutional delivery coverage for adolescents in 54 low- and middle-income countries, 2000–2030. BMC Med.

